# Looking forward to the next 15 years: innovation and new pathways for research in health equity

**DOI:** 10.1186/s12939-017-0531-0

**Published:** 2017-02-21

**Authors:** Ana Lorena Ruano, Efrat Shadmi, John Furler, Krishna Rao, Miguel San Sebastián, Manuela Villar Uribe, Leiyu Shi

**Affiliations:** 1 Center for the Study of Equity and Governance in Health Systems, CEGSS, Guatemala City, Guatemala; 2 Center for International Health, University of Bergen, Bergen, Norway; 3 The Cheryl Spencer Department of Nursing, Faculty of Socail Welfare and Health Sciences, the University of Haifa, Haifa, Israel; 4 Department of General Practice, Faculty of Medicine, Dentistry and Health Sciences University of Melbourne, Melbourne, Australia; 5 Johns Hopkins Bloomberg School of Public Health, Baltimore, Maryland USA; 6 Department of Public Health and Clinical Medicine, Unit of Epidemiology and Global Health Umeå University, SE-901 87 Umeå, Sweden; 7 Johns Hopkins Bloomberg School of Public Health, Baltimore, Maryland USA

**Keywords:** Equity, Indigenous peoples, Right to health, Refugees, Non-Nationals, SDGs, Qualitative methods, Social justice, Cooperation

## Abstract

Since our launch in 2002, the International Journal for Equity in Health (IJEqH) has furthered our collective understanding of equity in health and health services by providing a platform on which academics and practitioners can share their work. Today, we celebrate our fifteenth anniversary with an article collection that presents a call for new and novel research in equity in health and we invite our authors to use new approaches and methods, and to focus on emerging areas of research related to health equity in order to set the stage for the next fifteen years of health equity research.

Our anniversary issue provides a platform for expanding the conceptualization, diversity of populations and study designs, and for increasing the use of novel methodologies in the field. The IJEqH has helped to support the wider group of researchers, policymakers and practitioners with a commitment to social justice and equity but there is still more to do. With the help of the highly committed editorial team and editorial board, the innovative work of researchers worldwide, and the countless of hours dedicated by hundreds of reviewers, we are confident in the IJEqH’s ability to continue supporting the dissemination of health equity research for years to come.

Since our launch in 2002, the International Journal for Equity in Health (IJEqH) has furthered our collective understanding of equity in health and health services by providing a platform on which academics and practitioners can share their work. Our mandate continues to be the publication of political, policy-related, economic, social and health services research that focuses on the systematic differences in the distribution of one or more aspects of health in population groups defined demographically, geographically or socially. Our commitment to giving voice to authors from high-, middle- and low-income countries remains just as strong today as when Prof Barbara Starfield founded the journal fifteen years ago.

Understanding and acting on global and local inequity remains one of the greatest issues of our times, and our efforts to collect and publish research on particular topics of concern are reflected in our three long-running thematic series. The first of these, which focuses on multimorbidity (MM), studies the co-occurrence of health conditions in individuals and populations who experience high burdens of disease and who already find themselves in disadvantaged circumstances. The series is edited by Prof Efrat Shadmi, our co-Editor-in-Chief [1], and stems from Prof Starfield’s work showing that ‘only a person-focused (rather than a disease-focused) view of morbidity, in which multiple illnesses interact in myriad ways, can accurately depict the much greater impact of illness among socially disadvantaged people and the nature of the interventions that are required to adequately manage the increased vulnerability to and interactions among diseases’ [2]. The series contributes to the growing body of knowledge on MM and equity and presents an international perspective on the prevalence of MM in diverse population groups [3], its determinants [4] and outcomes [5].

Our second thematic series deals with the unprecedented economic crisis that has affected Europe, mainly in the southern countries, and is edited by our Associate Editor, Prof Miguel San Sebastián, and Guest Editor Dr Antonio Escolar-Pujolar. While certain macroeconomic recovery has been observed in the last couple of years, the economic situation remains unstable. This is particularly true for the Mediterranean countries, which still face numerous social and economic challenges [6]. This series aims to raise awareness of the impacts of the economic crisis on population health and, especially, on health inequalities. The articles within the series showcase the diversity and complexity of the impact of the crisis on health [7], but further studies are needed to better understand the link between health and the policies emanating from a dominant socioeconomic model that continuously generates socioeconomic inequalities that affect the most vulnerable groups in society [8].

Finally, our thematic series on interventions in primary health care that improve equity of outcomes in health showcases the potential contributions of continuous, comprehensive, and coordinated primary health care. Prof Starfield’s seminal work demonstrated that effective primary care is associated with improved access to health care services, reduced hospitalizations, cost-effectiveness and enhanced equity [9–12]. In recent years, many countries, organizations, communities, and world agencies have implemented primary care reforms and interventions to improve healthcare access, quality, health outcomes and equity in health. This series aims to create a space where we can document and analyze primary health care reforms and interventions that communities, organizations, countries and world agencies have undertaken [13] and is edited by our Associate Editor, Dr John Furler; our Managing Editor, Dr Ana Lorena Ruano; and Prof Leiyu Shi, co-Editor-in-Chief.

Today we celebrate our fifteenth anniversary with an article collection that presents a call for new and novel research in equity in health. The papers presented here invite our authors to use new approaches and methods, and to focus on emerging areas of research related to health equity. This is the case of the article by Fridman and Gostin [14], who use the Framework Convention on Global Health to call for a profound questioning of health policies at the global, national and local levels and who ask for research to guide actions geared towards truly reducing inequity in health through activism that can help reshape power dynamics. This is in line with Rasanathan and Diaz’s commentary [15], which focuses on Sustainable Development Goals (SDGs) and the need for strengthened development, testing, and implementation of possible solutions for improving equity levels around the globe. Brolan et al. [16] use a rights-based approach and state that the SDGs can be truly transformative if they are made operational in all countries and if they include nationals and non-nationals alike. This is especially urgent given the conflicts in the Middle East and Africa, as well as the escalating violence in Central America, which has forced hundreds of thousands of adults and unaccompanied minors to flee in perilous conditions and with little immediate hope of improving their lives and those of their loved ones.

Using systems thinking and engaging in complexity studies, Hernánez et al. [17] call for a novel approach to improving the health status of indigenous peoples, who remain among the most excluded and marginalized population groups all over the world [18]. The authors invite us to move from reductionist approaches that frame indigenous health as a set of poor health indicators and instead focus on holistic, integrated approaches that address the root causes of inequity both inside and outside the health sector. This approach raises the need to expand our methodological and theoretical toolbox, particularly when it comes to social sciences and qualitative approaches. Hannefeld et al., on behalf of the SHAPES thematic working group from Health Systems Global, also focus on the role of different methodologies and approaches to enriching our understanding of inequity [19]. Without a network of researchers of diverse backgrounds and methodological interests, our comprehension of issues that are central to improving the quality of health services and the tools we have at our disposal to strengthen health systems would be greatly diminished. Because of this, our anniversary issue enshrines our commitment to publish high-quality qualitative and social science research.

Social justice is at the heart of improving equity in health, and Devia et al. [20] examine the role of community-based participatory research in addressing the social determinants of health through working with the underlying causes of the inequitable distribution of resources and power structures. Flores et al. [21] show the urgent need for health professionals to be better equipped to address the cultural diversity of the populations seeking care in many countries today. Providing adequate support and training to new physicians and other health cadres can not only help ensure academic and professional success but also improve the quality and equity of care provided.

The last paper in our anniversary issue focuses on the role of competition and collaboration in achieving greater levels of health equity. Chang and Fraser [22] argue for an agency-based approach, and warn that a zero-sum mentality with respect to competition leads to ethically questionable and less effective cooperation. Market-driven approaches to healthcare contribute greatly to the growing inequities around the world.

Setting the stage for the next fifteen years of health equity research, our anniversary issue provides a platform for expanding the conceptualization, diversity of populations and study designs, and for increasing the use of novel methodologies in the field. Recent global changes that instigate major demographic [23] and economic transformations [7] call for expanding the role of research in illuminating injustice, developing and testing interventions to expand the evidence base on how to eliminate inequity in health and health care, and guiding policy that will lead to better outcomes for diverse population groups worldwide. Fifteen years on, much has been achieved. The journal has helped support the wider group of researchers, policymakers and practitioners with a commitment to social justice and equity. There is still more to do. With the help of the highly committed editorial team and editorial board, the innovative work of researchers worldwide, and the countless of hours dedicated by hundreds of reviewers, we are confident in the IJEqH’s ability to continue supporting the dissemination of health equity research for years to come.
